# Refining inflammatory profiles linked to cardiometabolic outcomes in people with HIV using recursive feature addition modelling

**DOI:** 10.1002/ctm2.70713

**Published:** 2026-06-11

**Authors:** Rachel MacCann, Dana Alalwan, Gurvin Saini, Alejandro Abner Garcia Leon, Jesvin Xavier, Matthew Hunter, Fiorina Rigonat, Polina Smolovyk, Maarten F. Schim van der Loeff, Neeltje Kootstra, Padraig McGettrick, Aoife G. Cotter, Alan Winston, Peter Reiss, Caroline Sabin, Patrick W. Mallon, the UPBEAT‐CAD, AIID and COBRA cohorts

**Affiliations:** ^1^ School of Medicine University College Dublin Dublin Ireland; ^2^ Department of Infectious Diseases St Vincent's University Hospital Dublin Ireland; ^3^ Centre for Experimental Pathogen Host Research University College Dublin Dublin Ireland; ^4^ Department of Infectious Diseases Public Health Service of Amsterdam Amsterdam The Netherlands; ^5^ Amsterdam Institute for Immunology and Infectious Diseases Amsterdam The Netherlands; ^6^ Department of Experimental Immunology, Amsterdam University Medical Centers University of Amsterdam Amsterdam The Netherlands; ^7^ Department of Infectious Diseases Mater Misericordiae University Hospital Dublin Ireland; ^8^ Department of Infectious Disease Imperial College London London UK; ^9^ Amsterdam UMC location University of Amsterdam, Global Health Amsterdam The Netherlands; ^10^ Amsterdam Institute for Global Health and Development Amsterdam The Netherlands; ^11^ Institute for Global Health University College London London UK

1

Dear Editor,

We applied a recursive feature addition (RFA) framework to refine inflammatory biomarker clustering associated with a composite vascular phenotype (CVP) and metabolic syndrome (MetS) in people with and without HIV. Expansion of predefined biomarker panels improved inflammatory phenotype resolution and strengthened associations with cardiometabolic disease burden across both phenotypes.

Cardiometabolic diseases, including cardiovascular disease (CVD) and MetS, are becoming increasingly common in people with HIV despite effective antiretroviral treatment (ART).^1^ Alongside ageing, lifestyle factors and ART exposure, chronic immune activation and systemic inflammation may contribute to this excess risk. However, conventional risk prediction tools do not account for HIV‐associated immune dysregulation, potentially limiting their accuracy in this population. Recent studies have applied biomarker clustering to identify inflammatory phenotypes associated with cardiometabolic outcomes in people with HIV.^2^, ^3^, ^4^ We previously described an RFA modelling strategy to identify informative biomarkers that improve inflammatory cluster discrimination in relation to a CVP.^5^ Here, we extend this framework to both CVP and MetS to refine inflammatory phenotypes and better characterise associated biological and clinical features.

Consenting participants with and without HIV from three different prospective, multicentre cohort studies were included (Table 1).^5^ CVD burden was assessed using a CVP, encompassing hypertension, myocardial infarction, stroke or transient ischaemic attack, coronary artery disease or peripheral vascular disease.^4^, ^6^ MetS was defined according to NCEP ATP III criteria, using BMI > 30 kg/m^2^ in place of waist circumference due to data availability.

**TABLE 1:** Baseline demographics of combined cohorts.

Characteristic	People without HIV, *N* = 90	People with HIV, *N* = 318
Cohort		
All‐Ireland Infectious Diseases Cohort (AIID)	0	179 (100%)
HIV‐Infected Individuals With Coronary Artery Disease (HIV UPBEAT CAD)	30 (46%)	35 (54%)
Co‐Morbidity in Relation to HIV/AIDS (COBRA)	61 (37%)	103 (63%)
Age (years), median (IQR)	55 (49, 63)	49 (40, 55)
Sex, male	83 (93%)	254 (80%)
Ethnicity		
Asian	1 (1.1%)	12 (3.8%)
Black	4 (4.5%)	68 (22%)
Hispanic/Brazilian	0 (0%)	44 (14%)
White	83 (94%)	192 (61%)
Viral suppression on ART (<200 cells/mm^3^)	NA	318 (100%)
CD4 count (cells/mm^3^), median (IQR)	NA	614 (481, 795)
Nadir CD4 count (cells/mm^3^), median (IQR)	NA	210 (104, 328)
ART duration (years), median (IQR)	NA	10 (6, 16)
MSM	48 (81%)	203 (64%)
Smoking status		
Current	22 (25%)	68 (22%)
Former smoker	29 (33%)	68 (22%)
Never smoked	37 (42%)	176 (56%)
Any CVP condition	38 (42%)	98 (31%)
Hypertension	30 (34%)	89 (28%)
Heart failure	2 (2.3%)	2 (.6%)
Stroke and/or transient ischaemic attack	0 (0%)	4 (1.3%)
Myocardial infarction	2 (2.2%)	1 (.3%)
Coronary artery disease	11 (12%)	12 (3.8%)
Peripheral vascular disease	0 (0%)	3 (.9%)
ASCVD risk score (%), median (IQR)	10 (4, 19)	5 (2, 12)
Metabolic syndrome	17 (19%)	66 (21%)
Elevated BMI (>30 kg/m^2^)	18 (20%)	71 (23%)
BP >140 mmHg or history of hypertension	37 (41%)	132 (42%)
History of diabetes/elevated glucose (>6.1 mmol/L)	18 (20%)	56 (18%)
Low HDL <1.03 in males, <1.29 in females (mmol/L)	22 (24%)	89 (28%)
Elevated triglycerides (>1.7 mmol/L)	34 (38%)	113 (36%)

*Note*: Values are presented as median (IQR) or *n* (%). Participants may be represented in more than one category as CVP categories may overlap.

Abbreviations: ART, antiretroviral therapy; ASCVD, atherosclerotic cardiovascular disease risk score; CVP, composite cardiovascular phenotype (hypertension, heart failure, myocardial infarction, transient ischaemic attack/cerebrovascular accident, or coronary/peripheral vascular disease); HDL, high‐density lipoprotein; IQR, interquartile range; MSM, men having sex with men.

Plasma concentrations of 55 biomarkers were quantified using multiplex immunoassay platforms and targeted single‐plex assays. Initial clusters were derived using previously validated biomarker panels associated with CVP (24 markers)^4^ and MetS (17 markers)^7^, ^8^, ^9^ using principal component analysis (PCA) and hierarchical clustering (HC) (Table S1). RFA modelling was applied to determine whether additional biomarkers improved cluster classification. This framework, including internal cross‐validation, feature selection and external validation procedures, has been described previously.^5^ Biomarkers were retained based on classification performance and permutation importance. Refined clusters were rederived using PCA and HC, and associations with CVP and MetS were assessed using logistic regression models adjusted for demographic and cardiometabolic risk factors. Interaction analyses assessed whether these cluster associations differed according to HIV status.

Among 408 participants, median age was 50 years (interquartile range [IQR] 43–58), 83% (*n* = 339) were male, 68% (*n* = 277) were White and 78% (*n* = 318) were living with HIV (Table 1). CVP was present in 33.3% (*n* = 136) while 20% (*n* = 83) met diagnostic criteria for MetS.

Using the initial 24‐biomarker panel associated with CVP, PCA and HC identified three inflammatory clusters (Figure 1a). One cluster demonstrated a pro‐inflammatory profile characterised by elevated markers of T‐cell differentiation (IL‐2, IL‐12, thymic stromal lymphopoietin [TSLP] and interferon‐γ [IFN‐γ]), innate immune activation (tumour necrosis factor‐alpha [TNF‐α] and macrophage inflammatory protein‐1α [MIP‐1α]), systemic inflammation (IL‐6 and IL‐1β) and coagulation (soluble CD40L) (Table S2), consistent with pathways implicated in chronic immune activation and vascular inflammation.^10^ Participants in this cluster had less favourable cardiometabolic profiles, including older age (median 51 years; IQR 46–55), higher BMI (27.6 kg/m^2^; IQR 24.6–29.9 kg/m^2^) and elevated triglycerides (1.97 mmol/L; IQR 1.21‒2.59) (Table S3). Cluster membership showed modest discrimination for prevalent CVP compared with the uninflamed reference cluster (adjusted odds ratio [aOR] 1.50;  .69‒3.18; *p* =  .15) (Figure 2).

**FIGURE 1:**
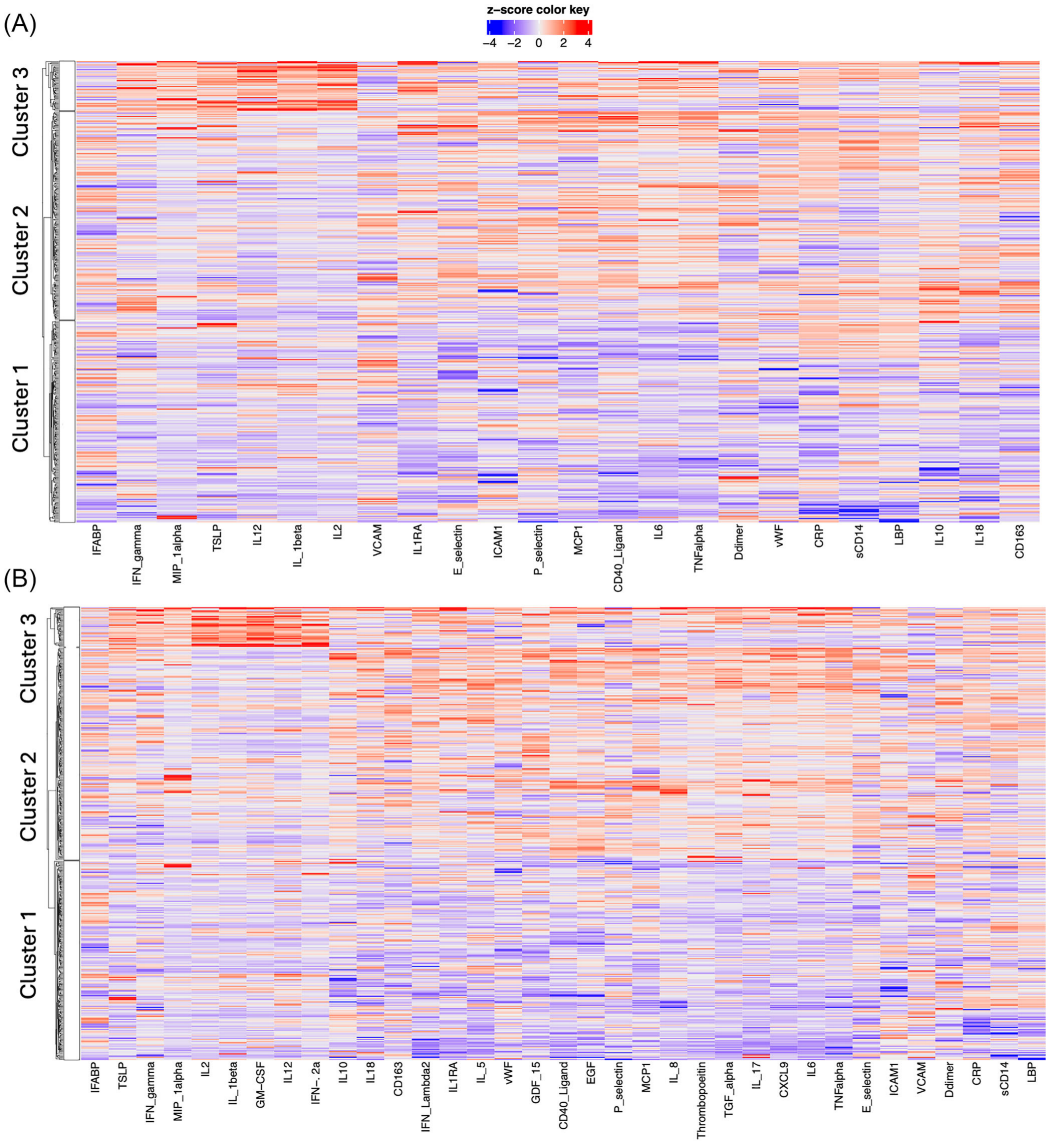
Recursive feature addition (RFA) refines inflammatory biomarker clustering associated with composite vascular phenotype (CVP). (A) Heatmap showing clustering of participants using the initial 24‐biomarker panel previously associated with cardiovascular disease in people with HIV. (B) Heatmap showing clustering after RFA, in which additional biomarkers were sequentially evaluated using a random forest classifier to improve cluster classification performance. Biomarker concentrations were log‐transformed, standardised and visualised as scaled values (*z*‐scores). Clustering was performed using principal component analysis followed by hierarchical clustering on principal components (Ward's method). Rows represent biomarkers and columns represent individual participants. Colours indicate relative biomarker expression (red = higher, blue = lower). Annotated bars indicate cluster membership and cardiovascular phenotype.

**FIGURE 2:**
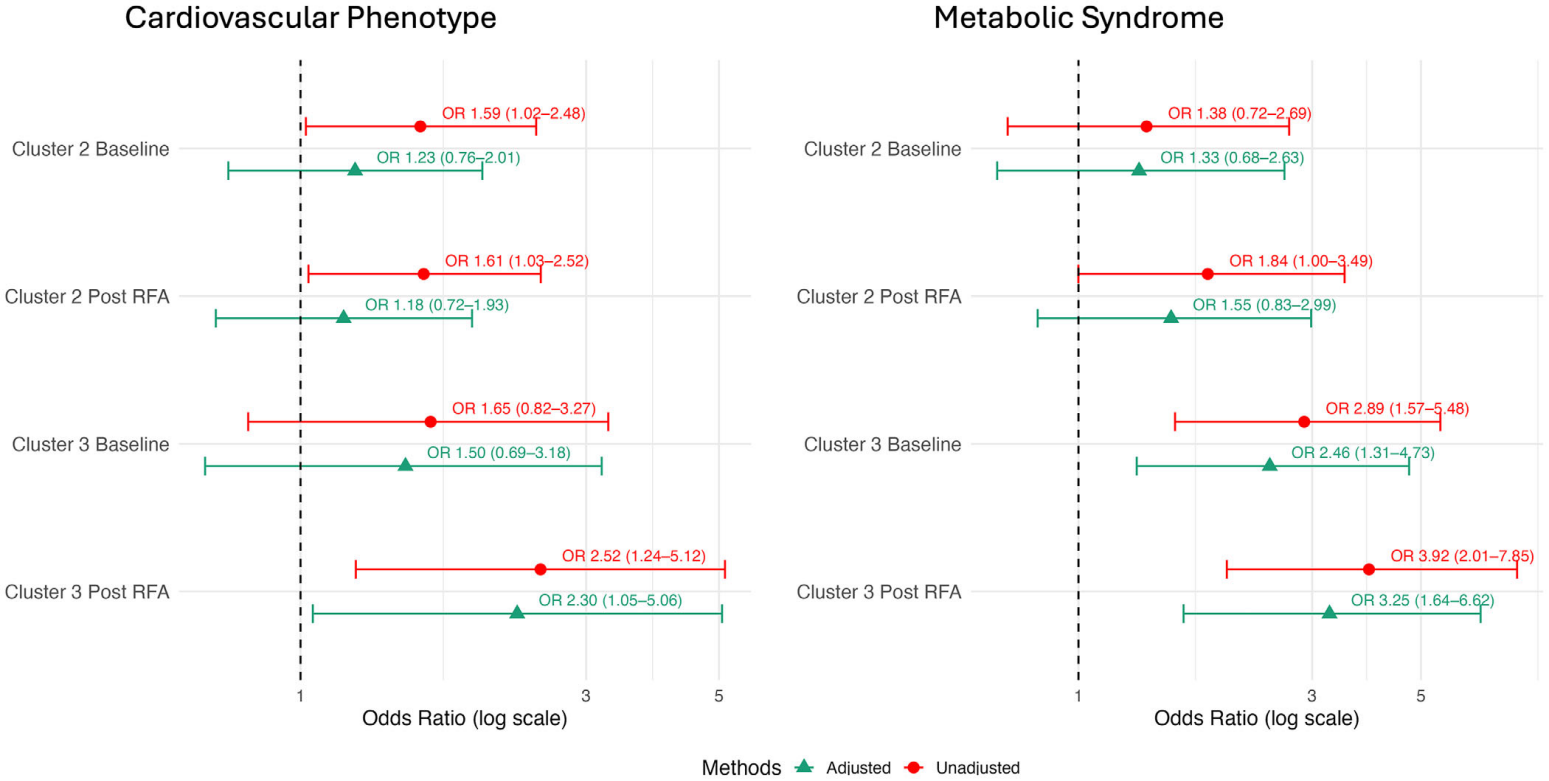
Forest plot showing unadjusted and adjusted logistic regression of relationship between cluster membership and non‐cummincable diseases, with cluster 1 as the reference cluster. Forest plots of unadjusted and adjusted odds ratios (ORs) for the cardiovascular phenotype (CVP) and metabolic syndrome (MetS) across clusters. Cardiovascular disease (CVD) models were adjusted for age, sex, smoking history, BMI and dyslipidaemia. MetS models were adjusted for age, sex, ethnicity and smoking. Error bars indicate 95% confidence intervals.

Following RFA, 11 additional biomarkers were identified and added to the original 24‐marker CVP panel. PCA and HC were then repeated using this expanded biomarker set, again resolving three inflammatory clusters, including a refined pro‐inflammatory cluster (Figure 1b). Compared with the initial analysis, this cluster showed elevated granulocyte–macrophage colony‐stimulating factor (GM‐CSF), IFN‐α2a, transforming growth factor‐α and thrombopoietin, broadening the represented biological pathways (Table S2). This refined cluster implicated chemotactic signalling, myeloid activation and platelet‐associated pathways linked to endothelial activation and vascular dysfunction. Participants within this refined inflammatory cluster demonstrated a more pronounced cardiometabolic profile and higher odds of CVP (aOR 2.30; 95% confidence interval [CI] 1.05–5.06; *p* = .037), suggesting improved discrimination of inflammatory phenotypes associated with vascular disease burden (Figure 2 and Table S4).

Applying the same analytical framework to MetS, clustering based on a predefined 17‐biomarker panel identified three inflammatory phenotypes (Figure 3a). A pro‐inflammatory cluster demonstrated elevations in systemic inflammation (CRP and IL‐6), innate immune activation (TNF‐α, MCP‐1 and CD163) and obesity‐related adipokines (leptin and resistin), consistent with pathways linking inflammation and metabolic dysfunction in people with and without HIV (Table S5) Compared with the uninflamed reference cluster, participants in this cluster were older (median 52 years; IQR 47–60), had higher BMI (27.8 kg/m^2^; IQR 23.4–30.8), and greater metabolic burden, with increased odds of MetS (aOR 2.46; 95% CI 1.31–4.73; *p* = .006) (Figure 2 and Table S6). RFA introduced eight additional biomarkers linked to interferon signalling, immune regulation and coagulation, including CXCL9, IFN‐λ2a, IL‐1RA and thrombopoietin (Figure 3b). Participants in this refined inflammatory cluster demonstrated a similar cardiometabolic profile to the initial model but showed stronger associations with MetS (aOR 3.25; 95% CI 1.64–6.62; *p* = .0027) (Table S7).

**FIGURE 3:**
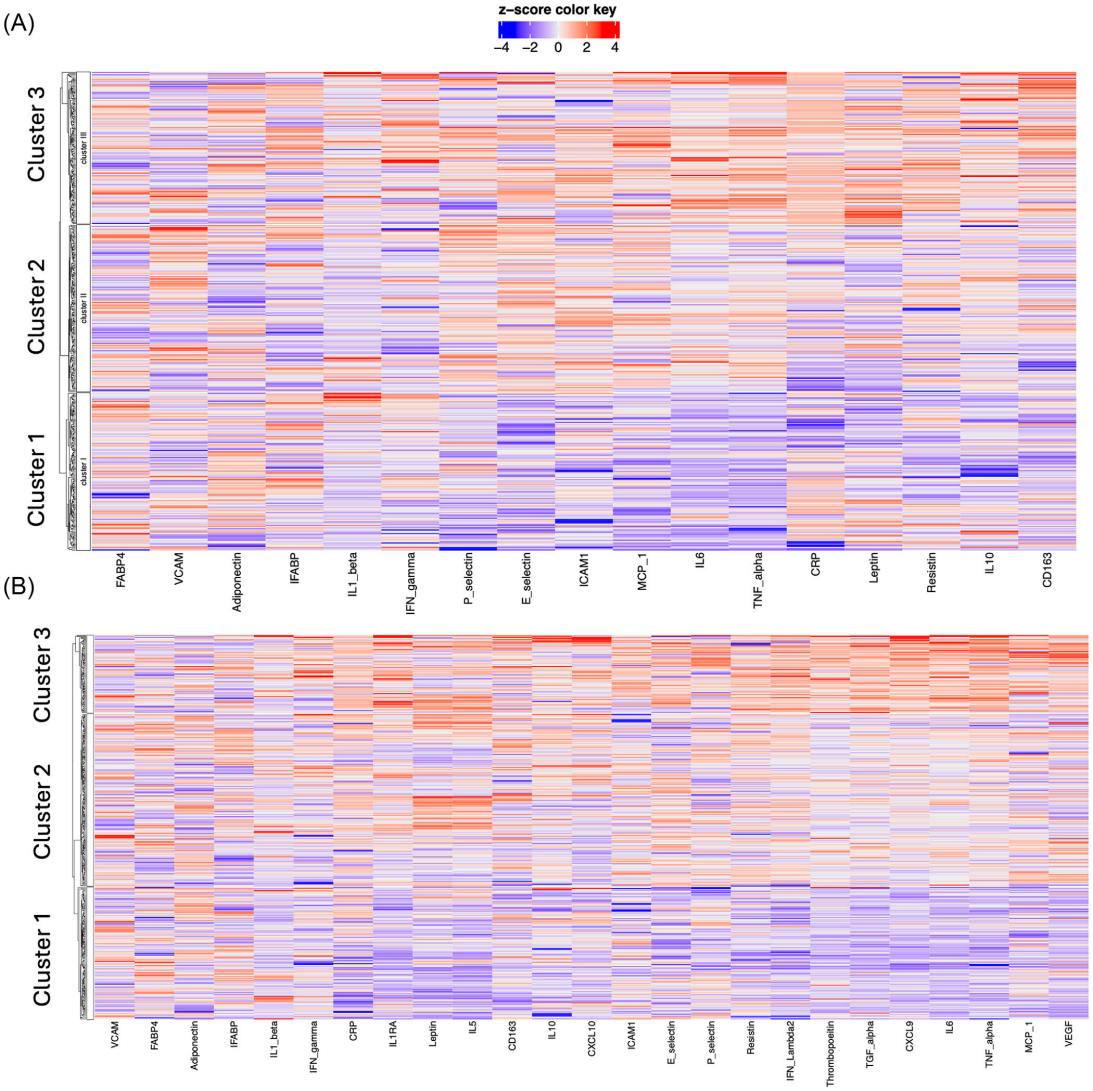
Recursive feature addition (RFA) refines inflammatory clusters associated with metabolic syndrome (MetS). (A) Heatmap generated using the predefined 17‐biomarker panel associated with metabolic syndrome. (B) Heatmap showing clusters after RFA, incorporating eight additional biomarkers that improved classification performance. Biomarkers were log‐transformed and scaled to unit variance prior to analysis. Clustering was performed using principal component analysis followed by hierarchical clustering on principal components. Colours represent relative biomarker abundance across participants.

Across both CVP and MetS models, HIV prevalence did not differ across clusters, although interaction analyses suggested stronger associations between the refined CVP inflammatory phenotype and vascular disease burden in HIV‐negative participants, with no interaction observed for MetS (Tables S8 and S9). Among people with HIV, inflamed cluster membership showed non‐significant trends towards longer ART exposure and lower nadir CD4 measures.

However, the cross‐sectional design, predominantly male and White study population and composite CVP outcome limit generalisability, causal inference and interpretation of individual cardiovascular associations, particularly given the inclusion of hypertension within the composite phenotype. Similar clustering patterns were observed in our prior methodological analysis when hypertension was excluded from the vascular phenotype definition,^5^ suggesting that these inflammatory associations were not solely driven by hypertension. Cohort heterogeneity and batch effects were addressed using harmonised preprocessing and batch‐correction approaches as previously described.^5^ Prospective, multi‐omic studies are required to further validate and determine the broader applicability of these findings.

In conclusion, the identification of additional inflammatory pathways across both CVP and MetS indicates that RFA may improve inflammatory phenotype resolution across cardiometabolic conditions. These findings suggest that inflammatory profiling may complement traditional clinical risk factors and contribute to more personalised cardiometabolic risk assessment in people with HIV.

## AUTHOR CONTRIBUTIONS

Rachel MacCann conceived the study, recruited participants from the AIID cohort, performed the statistical analyses and wrote the manuscript. Matthew Hunter contributed to data entry. Gurvin Saini and Jesvin Xavier performed the laboratory analysis. Alejandro Abner Garcia Leon contributed to the statistical analysis. Caroline Sabin, Alan Winston, Aoife G. Cotter, Peter Reiss, Neeltje Kootstra and Padraig McGettrick all oversaw participant recruitment and data acquisition. Caroline Sabin contributed to the design and analysis of the study. Patrick W. Mallon contributed to the study design, analysis and manuscript preparation. All the authors reviewed the final manuscript.

## CONFLICT OF INTEREST STATEMENT

A.C. has received honoraria, educational or travel support, and unrestricted research funding from Gilead Sciences, MSD, ViiV Healthcare and Janssen‐Cilag. A.W. has received honoraria or research funding through Imperial College London and has participated as a consultant or investigator in clinical trials sponsored by Bristol‐Myers Squibb, Gilead Sciences, GlaxoSmithKline, Janssen‐Cilag, Roche and ViiV Healthcare. P.W.G.M. has received honoraria and/or travel support from Gilead Sciences, MSD, Bristol‐Myers Squibb and ViiV Healthcare. P.R. has received independent scientific grant funding from Gilead Sciences, Janssen Pharmaceuticals, Inc., Merck & Co. and ViiV Healthcare, and has served on scientific advisory boards for Gilead Sciences, ViiV Healthcare and Merck & Co.; all honoraria were paid to his institution. C.S. has received honoraria for the preparation and delivery of educational materials from Gilead Sciences and ViiV Healthcare. The remaining authors declare they have no conflicts of interest.

## FUNDING INFORMATION

This work was performed within the Irish Clinical Academic Training Programme, supported by the Wellcome Trust and the Health Research Board (grant number 203930/B/16/Z), the Health Service Executive, National Doctors Training and Planning and the Health and Social Care, Research and Development Division, Northern Ireland. R.M.C. also received additional funding from the British HIV Association (BHIVA) from a 2022 BHIVA research award. The COBRA study was supported by a European Union's Seventh Framework Programme grant to the Comorbidity in Relation to AIDS (COBRA) project (FP‐7‐HEALTH 305,522), National Institute for Health Research (NIHR) Professorship (NIHR‐RP‐011‐048), NIHR Imperial Biomedical Research Centre, the Netherlands Organisation for Health Research and Development (grant number 300020007) and Stichting AIDS Funds (grant number 2009063), Nuts‐Ohra Foundation (grant number 1003–026) and investigator initiated grants from Gilead Sciences, ViiV Healthcare, Janssen Pharmaceutica N.V. Bristol‐Myers Squibb (BMS) and Merck & Co. to the AGEhIV cohort study, and investigator initiated grants from BMS, Gilead Sciences, Janssen, Merck and ViiV Healthcare to the POPPY cohort study. The HIV UPBEAT Cohort Study has received funding from the Health Research Board (award number HRA_POR/2010/66).

## ETHICS STATEMENT

Ethical approvals for the AIID, UPBEAT‐CAD and COBRA cohort studies were obtained from the relevant institutional review boards.^5^ All participants provided written informed consent.

## Supporting information



Supporting information

## Data Availability

Requests for data access will be considered on a case‐by‐case basis through the respective cohort investigators.
