# Supplementary material for: Refining inflammatory profiles linked to cardiometabolic outcomes in people with HIV using recursive feature addition modelling
Source: Clin Transl Med. 2026 Jun 11;16(6):e70713. doi: 10.1002/ctm2.70713 (PMC13260666; doi:10.1002/ctm2.70713)
Supplement: Supplementary file 1 — Supporting information [file CTM2-16-e70713-s001.docx]

# **Supplementary Data**

[Table S 1. Biomarker Panel by Pathway with Indication of Inclusion in Baseline and Post-RFA Models for the CVP and Metabolic Syndrome 2](#_Toc229147469)

[Table S 2 . Biomarkers defining cardiovascular disease (CVD) clusters before and after RFA modelling. 4](#_Toc229147470)

[Table S 3 Participant characteristics by cluster in the initial CVP model 5](#_Toc229147471)

[Table S 4. Participant characteristics by cluster in the CVP model post-RFA 6](#_Toc229147472)

[Table S 5 Biomarkers defining Metabolic Syndrome (MetS) clusters before and after RFA modelling. 7](#_Toc229147473)

[Table S 6. Demographics and MetS disease risk factors of participants defined by cluster allocation in the initial model 8](#_Toc229147474)

[Table S 7. Demographics and MetS disease risk factors of participants defined by cluster allocation post-RFA modelling and inclusion of additional inflammatory biomarkers. 9](#_Toc229147475)

[Table S 8. Associations between inflammatory clusters and prevalent cardiovascular disease, including HIV interaction and stratified analyses for the post-RFA model. 10](#_Toc229147476)

[Table S 9. Associations between inflammatory clusters and prevalent metabolic syndrome, including HIV interaction and stratified analyses for the post-RFA model. 11](#_Toc229147477)

**Table S 1. Biomarker Panel by Pathway with Indication of Inclusion in Baseline and Post-RFA Models for the CVP and Metabolic Syndrome**

| Pathway | Biomarker | CVP initial model | CVP post-RFA model | MetS initial model | MetS post-RFA model |
| --- | --- | --- | --- | --- | --- |
| Systemic inflammation | hsCRP | Y | Y | Y | Y |
|  | IL-6 | Y | Y | Y | Y |
|  | IL1β | Y | Y | Y | Y |
|  | IL-17 |  | Y |  |  |
|  | TNF- α | Y | Y | Y | Y |
|  | PDL-1 |  |  |  |  |
|  | Procalcitonin |  |  |  |  |
|  | IFN-γ | Y | Y | Y | Y |
|  | IL-5 |  | Y |  | Y |
|  | IL-7 |  |  |  |  |
|  | IL-8 |  | Y |  |  |
|  | GM-CSF |  | Y |  |  |
| T Helper 1 response | IL-2 | Y | Y |  |  |
|  | IL-18 | Y | Y |  |  |
|  | IL-12p70 | Y | Y |  |  |
| Innate Immune activation | sCD163 | Y | Y | Y | Y |
|  | MCP1 | Y | Y | Y | Y |
|  | MIP1a | Y | Y |  |  |
|  | MDC |  |  |  |  |
|  | CXCL9 |  | Y |  | Y |
|  | CXCL10 |  |  |  | Y |
| Microbial translocation | sCD14 | Y | Y |  |  |
|  | LBP | Y | Y |  |  |
|  | Zonulin |  |  |  |  |
|  | Beta-D-Glucan |  |  |  |  |
| Endothelial activation | ICAM-1 | Y | Y | Y | Y |
|  | VEGF |  |  |  | Y |
|  | E selectin | Y | Y | Y | Y |
|  | vWF | Y | Y |  |  |
|  | sVCAM-1 | Y | Y | Y | Y |
| Coagulation | P selectin | Y | Y | Y | Y |
|  | sCD40L | Y | Y |  |  |
|  | D Dimer | Y | Y |  |  |
|  | Thrombopoietin |  | Y |  | Y |
| T cell modulation | TSLP | Y | Y |  |  |
| Obesity | Adiponectin |  |  | Y | Y |
|  | Leptin |  |  | Y | Y |
|  | Resistin |  |  | Y | Y |
|  | FABP4 |  |  | Y | Y |
| Antiviral | IFN-α2a |  | Y |  | Y |
|  | IFN-β |  |  |  |  |
|  | IFN-λ1 |  |  |  |  |
|  | IFN-λ2 |  | Y |  |  |
|  | IFN-λ3 |  |  |  |  |
| Tissue repair | EGF |  | Y |  |  |
|  | TGF- α |  | Y |  | Y |
|  | PDGF-AA |  |  |  |  |
| Immune regulation | IL-33 |  |  |  |  |
|  | IL-15 |  |  |  |  |
|  | IL-1RA | Y | Y |  | Y |
| Anti-inflammatory | IL-10 | Y | Y | Y | Y |
|  | GDF-15 |  | Y |  |  |
|  | IL-13 |  |  |  |  |
| Neuronal Pathway | Beta-NGF |  |  |  |  |
| Gut epithelial barrier disruption | I-FABP | Y | Y | Y | Y |

CXCL, C-X-C motif chemokine ligand; EGF, Epidermal Growth Factor; E-selectin, Endothelial-Selectin; GM-CSF, Granulocyte-Macrophage Colony-Stimulating Factor; GP-6, Glycoprotein VI; GDF-15, Growth Differentiation Factor 15; hsCRP, high sensitivity C Reactive protein; IFN, Interferon; I-FABP, intestinal fatty acid binding protein; IL, Interleukin; IL1b, interleukin 1 beta; IL1RA, IL-1 receptor antagonist; LBP, LPS binding protein; MCP-1, Monocyte chemoattractant protein; MDC, Macrophage-Derived Chemokine; MIP-1, macrophage inflammatory protein; PDGF-AA, Platelet-Derived Growth Factor-AA; PD-L1, programmed death-ligand 1; P-selectin, Platelet-Selectin; RANTES, Regulated upon Activation, Normal T cell Expressed and Secreted; sCD163, soluble cluster of differentiation 163; sCD40L, soluble CD40 ligand; s-ICAM, soluble intercellular adhesion molecule; sVCAM-1, soluble Vascular Cell Adhesion Molecule-1; TNF-α, tumour necrosis factor alpha; TNFR, tumour necrosis factor receptor; TGF-α, Transforming Growth Factor-alpha; TSLP, Thymic Stromal Lymphopoietin; VCAM1, Vascular cell adhesion molecule; VEGF, Vascular Endothelial Growth Factor; vwf, von Willebrand factor.

**Table S 2 . Biomarkers defining cardiovascular disease (CVD) clusters before and after RFA modelling.**

| Cluster | Biomarker characterisations | Baseline 24 biomarkers | post RFA model |
| --- | --- | --- | --- |
| 1  Uninflamed | Low | TNF- α, IL-6, CD40-Ligand, IL-18, IL1RA, MCP-1, E-selectin, CD163, vWF and CRP | TNF- α, CD40-Ligand, CXCL9, IL6, TGF- α, IFN-Lambda2, IL1RA, IL-17, GDF-5 and IL-8 |
| 2  Vascular/ endothelial inflamed | High | TNF- α, CD40-Ligand, IL-18, IL-6, MCP-1, E-selectin, vWF, P-selectin, CD163 and IL-10 | TNF- α, CD40-Ligand, CXCL9, IFN-Lambda2, EGF, TGF- α, IL-17, IL-6, MCP1 and IL-5 |
|  | Low | IL-2, IL-12 and IL-1beta | IL-12, GM-CSF, IFN-α2a and IL2 |
| 3  Pro-inflammatory | High | IL-2, IL-1beta, IL-12, TSLP, MIP-1 α, IFN-gamma, IL1RA, IL-6, TNF- α and CD40-Ligand | GM-CSF, IL-2, IL-1beta, IFN-α2a, IL-12, TSLP, IFN-gamma, MIP-1 α, IL1RA and IL-6 |

Key biomarkers defining each CVP cluster are shown at baseline and after RFA refinement. Following PCA and hierarchical clustering, each biomarker’s contribution as the standardised difference between its cluster mean and overall mean was quantified. Biomarkers with the largest differences were considered most influential and ranked to highlight the cluster-specific inflammatory profile. High/Low indicates relative expression within each cluster.

**Table S 3 Participant characteristics by cluster in the initial CVP model**

| **Characteristic** | **1 N = 179^1^** | **2 N = 185^1^** | **3 N = 44^1^** | **p-value^2^** |
| --- | --- | --- | --- | --- |
| Age | 49 (40, 55) | 51 (44, 61) | 51 (46, 55) | 0.026 |
| Sex, Male | 143 (81%) | 159 (86%) | 35 (80%) | 0.35 |
| Ethnicity |  |  |  | 0.14 |
| Asian | 8 (4.5%) | 5 (2.7%) | 0 (0%) |  |
| Black | 38 (22%) | 30 (16%) | 4 (9.3%) |  |
| Latino | 23 (13%) | 17 (9.2%) | 4 (9.3%) |  |
| White | 107 (61%) | 133 (72%) | 35 (81%) |  |
| Smoking |  |  |  | 0.53 |
| Current | 34 (20%) | 46 (25%) | 10 (23%) |  |
| Former smoker | 39 (23%) | 49 (26%) | 9 (21%) |  |
| Never smoked | 99 (58%) | 90 (49%) | 24 (56%) |  |
| MSM | 107 (65%) | 122 (71%) | 22 (58%) | 0.26 |
| BMI | 25.5 (23.4, 28.7) | 26.4 (23.2, 29.7) | 27.6 (24.6, 29.9) | 0.12 |
| Geographical location |  |  |  | 0.011 |
| Amsterdam | 54 (30%) | 51 (28%) | 7 (16%) |  |
| Dublin | 113 (63%) | 103 (56%) | 28 (64%) |  |
| London | 12 (6.7%) | 31 (17%) | 9 (20%) |  |
| History of Dyslipidaemia | 71 (40%) | 90 (49%) | 16 (36%) | 0.15 |
| CVP Components | 50 (28%) | 69 (38%) | 17 (39%) | 0.091 |
| Hypertension | 43 (24%) | 61 (33%) | 15 (35%) | 0.13 |
| Heart failure | 2 (1.1%) | 2 (1.1%) | 0 (0%) | >0.99 |
| TIA/CVA | 3 (1.7%) | 0 (0%) | 1 (2.3%) | 0.13 |
| Myocardial Infarction | 0 (0%) | 3 (1.6%) | 0 (0%) | 0.34 |
| CAD/ PVD | 11 (6.2%) | 12 (6.5%) | 3 (6.8%) | >0.99 |
| Circulating Lipid Measures |  |  |  |  |
| Total Cholesterol (mmol/L) | 5.10 (4.80, 5.60) | 5.10 (4.70, 5.60) | 5.20 (4.70, 6.20) | 0.71 |
| Low density lipoprotein (mmol/L) | 3.00 (2.36, 3.61) | 2.80 (2.10, 3.38) | 2.95 (2.27, 3.45) | 0.024 |
| High density lipoprotein (mmol/L) | 1.33 (1.08, 1.56) | 1.22 (1.03, 1.47) | 1.21 (1.00, 1.38) | 0.018 |
| Triglycerides (mmol/L) | 1.23 (0.90, 1.80) | 1.47 (0.90, 2.11) | 1.97 (1.21, 2.59) | <0.001 |
| ASCVD Risk Score (%) | 5 (2, 12) | 8 (3, 15) | 7 (3, 14) | 0.043 |
| HIV parameters |  |  |  | 0.36 |
| Living with HIV | 134 (75%) | 150 (81%) | 34 (77%) |  |
| ART Duration (years) | 10 (6, 15) | 11 (6, 16) | 12 (5, 20) | 0.67 |
| CD4 Count (cells/mm^3^) | 636 (483, 833) | 604 (474, 766) | 576 (487, 762) | 0.34 |
| Nadir CD4 count (cells/mm^3^) | 250 (129, 374) | 195 (80, 280) | 224 (142, 353) | 0.039 |
|  | | | | |
| ^1^Median (IQR); n (%),^2^Wilcoxon rank sum test; Fisher's exact test; Pearson's Chi-squared test  ASCVD, Atherosclerotic Cardiovascular Disease risk; ; ART: antiretroviral therapy; BMI: body mass index; CAD: coronary artery disease; IQR; interquartile range; MSM: Men who have sex with men; TIA: transient ischaemic attack. CVP categories are not mutually exclusive; participants may be represented in more than one category. | | | | |

**Table S 4. Participant characteristics by cluster in the CVP model post-RFA**

| **Characteristic** | **1 N = 180^1^** | **2 N = 192^1^** | **3 N = 36^1^** | **p-value^2^** |
| --- | --- | --- | --- | --- |
| Age | 49 (38, 55) | 51 (45, 60) | 54 (47, 58) | 0.006 |
| Sex, Male | 146 (82%) | 162 (84%) | 29 (81%) | 0.77 |
| Ethnicity |  |  |  | 0.19 |
| Asian | 9 (5.1%) | 4 (2.1%) | 0 (0%) |  |
| Black | 35 (20%) | 34 (18%) | 3 (8.6%) |  |
| Latino | 23 (13%) | 17 (8.9%) | 4 (11%) |  |
| White | 110 (62%) | 137 (71%) | 28 (80%) |  |
| Smoking |  |  |  | 0.71 |
| Current | 35 (20%) | 46 (24%) | 9 (26%) |  |
| Former smoker | 39 (23%) | 50 (26%) | 8 (23%) |  |
| Never smoked | 99 (57%) | 96 (50%) | 18 (51%) |  |
| MSM | 111 (66%) | 120 (67%) | 20 (65%) | 0.95 |
| BMI (kg/m^2^) | 25.0 (23.3, 28.5) | 26.9 (23.3, 29.9) | 27.4 (23.5, 29.2) | 0.11 |
| History of Dyslipidaemia | 69 (39%) | 94 (49%) | 14 (39%) | 0.12 |
| CVP Components |  |  |  |  |
| Hypertension | 42 (24%) | 64 (34%) | 13 (37%) | 0.066 |
| Heart failure | 3 (1.7%) | 1 (0.5%) | 0 (0%) | 0.56 |
| TIA/ CVA | 3 (1.7%) | 0 (0%) | 1 (2.8%) | 0.071 |
| Myocardial infarction | 1 (0.6%) | 2 (1.0%) | 0 (0%) | >0.99 |
| CAD/PVD | 12 (6.7%) | 11 (5.7%) | 3 (8.3%) | 0.76 |
| Circulating Lipid Measures | 51 (28%) | 70 (36%) | 15 (42%) | 0.14 |
| Total Cholesterol (mmol/L) | 5.10 (4.40, 5.80) | 4.90 (4.13, 5.50) | 4.90 (4.20, 5.70) | 0.21 |
| Low density lipoprotein (mmol/L) | 3.00 (2.30, 3.61) | 2.80 (2.15, 3.31) | 2.90 (2.27, 3.45) | 0.11 |
| High density lipoprotein (mmol/L) | 1.33 (1.06, 1.58) | 1.22 (1.03, 1.46) | 1.20 (0.96, 1.38) | 0.009 |
| Triglycerides (mmol/L) | 1.21 (0.86, 1.75) | 1.50 (0.96, 2.17) | 1.97 (1.20, 2.48) | <0.001 |
| ASCVD Risk score (%) | 5 (2, 11) | 8 (3, 15) | 9 (3, 14) | 0.011 |
| HIV related parameters |  |  |  |  |
| Living with HIV | 134 (74%) | 155 (81%) | 29 (81%) | 0.32 |
| ART Duration (years) | 10 (6, 15) | 11 (6, 17) | 15 (4, 21) | 0.15 |
| CD4 Count (cells/mm^3^) | 620 (484, 825) | 618 (476, 793) | 580 (466, 772) | 0.73 |
| Nadir CD4 count (cells/mm^3^) | 240 (129, 374) | 195 (80, 290) | 238 (128, 335) | 0.042 |
| ^1^Median (IQR); n (%), ^2^Wilcoxon rank sum test; Fisher's exact test; Pearson's Chi-squared test  ASCVD, Atherosclerotic Cardiovascular Disease risk; ART: antiretroviral therapy; BMI: body mass index; CAD: coronary artery disease; IQR; interquartile range; MSM: Men who have sex with men; TIA: transient ischaemic attack. CVP categories are not mutually exclusive; participants may be represented in more than one category. | | | | |

**Table S 5 Biomarkers defining Metabolic Syndrome (MetS) clusters before and after RFA modelling.**

| **Cluster** | **Biomarker characterisations** | **Baseline 17 biomarkers** | **Biomarkers post RFA model** |
| --- | --- | --- | --- |
| Inlfamed Cluster | High | TNF- α, CD163, IL-6, CRP, Leptin, Resistin, IL-10, MCP-1, IFN-γ, E-selectin | CXCL-9, TNF- α, IL-6, TGF- α, IL-1RA, IFN-λ2, Thrombopoietin, VEGF, MCP-1, IL-5 |
|  | Low | VCAM |  |
| Uninflamed Cluster | High |  |  |
|  | Low | TNF-alpha, IL6, E- selectin, MCP1, P-selectin, ICAM1, IL10, CD163, Resistin and CRP | TNF- α, IL-6, TGF- α, IL-1RA, IFN-λ2, Thrombopoietin, VEGF, MCP-1, IL-5, CXCL9, E-selectin, Leptin |
| Mixed inflamed cluster | High | E-selectin, P-selectin, ICAM1, VCAM and MCP1 | IL-5, Leptin, E-selectin, CRP, IFN-λ2, IL-10, IL-1RA |
|  | Low | Leptin, IFN-gamma, CRP, IFABP, CD163, Adiponectin and IL1_beta |  |

Key biomarkers defining each MetS cluster are shown at baseline and after RFA refinement. Following PCA and hierarchical clustering, each biomarker’s contribution as the standardised difference between its cluster mean and overall mean was quantified. Biomarkers with the largest differences were considered most influential and ranked to highlight the cluster-specific inflammatory profile. High/Low indicates relative expression within each cluster.

**Table S 6. Demographics and MetS disease risk factors of participants defined by cluster allocation in the initial model**

| **Characteristic** | **1, N = 135^1^** | **2, N = 143^1^** | **3, N = 130^1^** | **p-value^2^** |
| --- | --- | --- | --- | --- |
| **Age (years), median (IQR)** | 48 (38, 55) | 51 (43, 59) | 52 (47, 60) | <0.001 |
| **Sex, Male** | 105 (78%) | 128 (91%) | 104 (80%) | 0.009 |
| **Ethnicity** |  |  |  |  |
| Asian | 5 (3.8%) | 8 (5.7%) | 0 (0%) |  |
| Black | 29 (22%) | 19 (13%) | 24 (18%) |  |
| Latino | 19 (14%) | 17 (12%) | 8 (6.2%) |  |
| White | 80 (60%) | 97 (69%) | 98 (75%) |  |
| **Smoking** |  |  |  | 0.34 |
| Current | 27 (20%) | 36 (26%) | 27 (21%) |  |
| Former smoker | 28 (21%) | 31 (22%) | 38 (29%) |  |
| Never smoked | 78 (59%) | 71 (51%) | 64 (50%) |  |
| **Metabolic Syndrome** | 18 (13%) | 25 (17%) | 40 (31%) | 0.001 |
| Obesity (BMI >30 kg/m^2^) | 26 (20%) | 25 (19%) | 38 (31%) | 0.043 |
| BP >140 mmHg or History of Hypertension | 49 (36%) | 55 (38%) | 65 (50%) | 0.052 |
| History of Diabetes or elevated Glucose (>6.1 mmol/L) | 20 (15%) | 26 (18%) | 28 (22%) | 0.36 |
| Low HDL (<1.03 in Males, <1.29 in Females(mmol/L)) | 35 (26%) | 30 (21%) | 46 (35%) | 0.026 |
| Elevated Triglycerides (>1.7 mmol/L) | 35 (26%) | 48 (34%) | 64 (49%) | <0.001 |
| **HIV Parameters** |  |  |  |  |
| Living with HIV | 105 (78%) | 106 (74%) | 107 (82%) | 0.27 |
| ART Duration (years), median (IQR) | 10 (6, 15) | 9 (4, 16) | 13 (6, 18) | 0.057 |
| CD4 Count (cells/mm^3^), median (IQR) | 654 (489, 831) | 573 (476, 748) | 617 (476, 805) | 0.36 |
| Nadir CD4 count (cells/mm^3^), median (IQR) | 260 (155, 373) | 205 (79, 280) | 200 (100, 302) | 0.049 |
| ^1^Median (IQR); n (%) | | | | |
| ^2^Wilcoxon rank sum test; Fisher's exact test; Pearson's Chi-squared test | | | | |
| ART: antiretroviral therapy; BMI: body mass index; CAD: coronary artery disease; IQR; interquartile range; MSM: Men who have sex with men; | | | | |

**Table S 7. Demographics and MetS disease risk factors of participants defined by cluster allocation post-RFA modelling and inclusion of additional inflammatory biomarkers.**

| **Characteristic** | **1, N = 141^1^** | **2, N = 184^1^** | **3, N = 83^1^** | **p-value^2^** |
| --- | --- | --- | --- | --- |
| **Age (years), median (IQR)** | 47 (36, 54) | 51 (45, 59) | 53 (45, 61) | <0.001 |
| **Sex, Male** | 121 (86%) | 146 (80%) | 70 (84%) | 0.39 |
| **Ethnicity** |  |  |  |  |
| Asian | 8 (5.8%) | 4 (2.2%) | 1 (1.2%) |  |
| Black | 23 (17%) | 36 (20%) | 13 (16%) |  |
| Latino | 25 (18%) | 9 (4.9%) | 10 (12%) |  |
| White | 83 (60%) | 133 (73%) | 59 (71%) |  |
| **Smoking** |  |  |  | 0.58 |
| Current | 29 (21%) | 44 (24%) | 17 (21%) |  |
| Former smoker | 28 (20%) | 47 (26%) | 22 (27%) |  |
| Never smoked | 80 (58%) | 90 (50%) | 43 (52%) |  |
| **Metabolic Syndrome** | 17 (12%) | 37 (20%) | 29 (35%) | <0.001 |
| Obesity (BMI >30 kg/m^2^) | 24 (18%) | 41 (23%) | 24 (31%) | 0.038 |
| BP >140 mmHg or History of Hypertension | 50 (35%) | 77 (42%) | 42 (51%) | 0.084 |
| History of Diabetes or elevated Glucose (>6.1 mmol/L) | 20 (14%) | 32 (17%) | 22 (27%) | 0.065 |
| Low HDL (<1.03 in Males, <1.29 in Females(mmol/L)) | 37 (26%) | 45 (24%) | 29 (35%) | 0.19 |
| Elevated Triglycerides (>1.7 mmol/L) | 34 (24%) | 74 (40%) | 39 (47%) | <0.001 |
| **HIV Parameters** |  |  |  |  |
| People with HIV | 110 (78%) | 143 (78%) | 65 (78%) | >0.99 |
| ART Duration (years), median (IQR) | 10 (6, 15) | 11 (6, 16) | 11 (6, 18) | 0.50 |
| CD4 Count (cells/mm^3^), median (IQR) | 620 (482, 772) | 618 (496, 846) | 557 (443, 767) | 0.29 |
| Nadir CD4 count (cells/mm^3^), median (IQR) | 250 (121, 374) | 206 (107, 301) | 185 (90, 304) | 0.17 |
| ^1^Median (IQR); n (%) | | | | |
| ^2^Wilcoxon rank sum test; Fisher's exact test; Pearson's Chi-squared test | | | | |
| ART: antiretroviral therapy; BMI: body mass index; CAD: coronary artery disease; IQR; interquartile range; MSM: Men who have sex with men; | | | | |

**Table S 8. Associations between inflammatory clusters and prevalent cardiovascular disease, including HIV interaction and stratified analyses for the post-RFA model.**

| **Model** | **term** | **estimate** | **CI low** | **CI high** | **P value** |
| --- | --- | --- | --- | --- | --- |
| Interaction | Cluster 2 vs Cluster 1 | 1.35 | 0.49 | 3.77 | 0.562 |
| Interaction | Cluster 3 vs Cluster 1 | 9.49 | 2.15 | 52.37 | 0.00469 |
| Interaction | Cluster 2 × People with HIV | 0.85 | 0.26 | 2.78 | 0.788 |
| Interaction | Cluster 3 × People with HIV | 0.14 | 0.02 | 0.83 | 0.0364 |
| People with HIV | Cluster 2 vs Cluster 1 | 1.12 | 0.62 | 2.02 | 0.706 |
| People with HIV | Cluster 3 vs Cluster 1 | 1.3 | 0.48 | 3.41 | 0.598 |
| People without HIV | Cluster 2 vs Cluster 1 | 1.08 | 0.36 | 3.14 | 0.892 |
| People without HIV | Cluster 3 vs Cluster 1 | 6.28 | 1.36 | 36.37 | 0.025 |

Odds ratios (ORs) and 95% confidence intervals (CI) from logistic regression models evaluating the association between cluster membership and prevalent CVD. The “Interaction” model includes HIV status and cluster × HIV interaction terms; the cluster main effects represent associations among HIV-negative participants, and interaction terms indicate how these associations differ in people with HIV. Stratified models show ORs for clusters separately among people with and without HIV. All models are adjusted for age, sex, smoking history, body mass index, and dyslipidaemia.

**Table S 9. Associations between inflammatory clusters and prevalent metabolic syndrome, including HIV interaction and stratified analyses for the post-RFA model.**

| **Model** | **term** | **estimate** | **CI low** | **CI high** | **P value** |
| --- | --- | --- | --- | --- | --- |
| Interaction Model | Cluster 2 vs Cluster 1 | 2.86 | 0.77 | 13.88 | 0.141 |
| Interaction Model | Cluster 3 vs Cluster 1 | 2.87 | 0.55 | 16.5 | 0.208 |
| Interaction Model | People with HIV | 1.9 | 0.55 | 8.91 | 0.353 |
| Interaction Model | Cluster 2 × People with HIV | 0.45 | 0.08 | 2.01 | 0.315 |
| Interaction Model | Cluster 3 × People with HIV | 1.14 | 0.17 | 7.04 | 0.89 |
| People with HIV | Cluster 2 vs Cluster 1 | 1.29 | 0.63 | 2.72 | 0.487 |
| People with HIV | Cluster 3 vs Cluster 1 | 3.3 | 1.53 | 7.35 | 0.00271 |
| People without HIV | Cluster 2 vs Cluster 1 | 2.5 | 0.64 | 12.48 | 0.214 |
| People without HIV | Cluster 3 vs Cluster 1 | 2.58 | 0.47 | 15.53 | 0.272 |

Odds ratios (ORs) and 95% confidence intervals (CI) from logistic regression models evaluating the association between cluster membership and prevalent CVD. The “Interaction” model includes HIV status and cluster × HIV interaction terms; the cluster main effects represent associations among HIV-negative participants, and interaction terms indicate how these associations differ in people with HIV. Stratified models show ORs for clusters separately among people with and without HIV. All models are adjusted for age, sex, smoking history and race.
